# Patterns of Longitudinal Neural Activity Linked to Different Cognitive Profiles in Parkinson's Disease

**DOI:** 10.3389/fnagi.2016.00275

**Published:** 2016-11-23

**Authors:** Atsuko Nagano-Saito, Mohamed S. Al-Azzawi, Alexandru Hanganu, Clotilde Degroot, Béatriz Mejia-Constain, Christophe Bedetti, Anne-Louise Lafontaine, Valérie Soland, Sylvain Chouinard, Oury Monchi

**Affiliations:** ^1^Centre de Recherche de l'Institut Universitaire de Gériatrie de MontréalMontréal, QC, Canada; ^2^Departments of Clinical Neurosciences and Radiology, University of CalgaryCalgary, AB, Canada; ^3^Hotchkiss Brain InstituteCalgary, AB, Canada; ^4^Department of Neurology and Neurosurgery, McGill UniversityMontreal, QC, Canada; ^5^Centre Hospitalier de l'Université de MontréalMontréal, QC, Canada

**Keywords:** Parkinson's disease, functional magnetic resonance image, Wisconsin Card Sorting Task, mild cognitive impairment, longitudinal study

## Abstract

Mild cognitive impairment in Parkinson's disease (PD) has been linked with functional brain changes. Previously, using functional magnetic resonance imaging (fMRI), we reported reduced cortico-striatal activity in patients with PD who also had mild cognitive impairment (MCI) vs. those who did not (non-MCI). We followed up these patients to investigate the longitudinal effect on the neural activity. Twenty-four non-demented patients with Parkinson's disease (non-MCI: 12, MCI: 12) were included in the study. Each participant underwent two fMRIs while performing the Wisconsin Card Sorting Task 20 months apart. The non-MCI patients recruited the usual cognitive corticostriatal loop at the first and second sessions (Time 1 and Time 2, respectively). However, decreased activity was observed in the cerebellum and occipital area and increased activity was observed in the medial prefrontal cortex and parietal lobe during planning set-shift at Time 2. Increased activity in the precuneus was also demonstrated while executing set-shifts at Time 2. The MCI patients revealed more activity in the frontal, parietal and occipital lobes during planning set-shifts, and in the parietal and occipital lobes, precuneus, and cerebellum, during executing set-shift at Time 2. Analysis regrouping of both groups of PD patients revealed that hippocampal and thalamic activity at Time 1 was associated with less cognitive decline over time. Our results reveal that functional alteration along the time-points differed between the non-MCI and MCI patients. They also underline the importance of preserving thalamic and hippocampal function with respect to cognitive decline over time.

## Introduction

In Parkinson's disease (PD), cognitive deficits are frequently present even early in the course of disease development (Foltynie et al., 2004). Mild cognitive impairment (MCI) has been conceptualized as a stage when cognitive deficits don't impede on daily activities (Litvan et al., 2012). The prevalence of MCI in the early stages of PD is estimated to be between 25 and 40% (Dalrymple-Alford et al., 2011). The evolution of cognitive deficits in PD remain poorly understood and medication treatment of cognitive deficits in PD yields very modest results (Goldman and Holden, 2014). Using functional Magnetic Resonance Imaging (fMRI) we recently studied the effect of MCI in PD on the patterns of fronto-striatal activations while performing the Wisconsin Card Sorting Task (WCST). When planning a set-shift in our WCST PD patients without MCI (PD non-MCI) revealed patterns of activation similar to healthy individuals in our previous studies (Monchi et al., 2004, 2007), with significant activation in the ventrolateral prefrontal cortex (PFC) and caudate nucleus. In contrast, PD patients with MCI (PD-MCI) had no significant activation in these regions (Nagano-Saito et al., 2014). Similar results have been reported by other studies that analyzed resting state networks (Baggio et al., 2015) or fMRI in PD-MCI and PD non-MCI patients while performing a working memory task (Lewis et al., 2003; Ekman et al., 2012; Nagano-Saito et al., 2014), suggesting that PD non-MCI have compensational patterns that allow them to maintain the cognitive function at the same level as healthy individuals, while MCI in PD is associated with the loss of compensational patterns and development of specific functional brain abnormalities in the cognitive cortico-striatal loops with decreased cortico-cortical and cortico-subcortical connectivity compared to PD non-MCI, including the caudate nucleus and the PFC. This hypothesis has been suggested by a previous longitudinal study using Positron Emission Tomography in PD patients while performing a visual sequence learning-task (Carbon et al., 2010). They observed that while hippocampus was not significantly solicited in healthy individuals, the PD patients who did decline in their performance between the baseline and repeated measurement, showed significantly increased regional cerebral blood flow in the hippocampus. In order to confirm whether functional patterns from our previous study are specific for MCI in PD, a longitudinal fMRI study has to be performed, that would help us understand the functional changes in these loops over time.

The aim of the present study was to use fMRI to longitudinally follow up the brain neural activity of non-demented PD patients with and without MCI while they were performing the WCST. We also aimed to find out which patterns of neural activity at Time 1 would be the most predictive of cognitive decline over time. We applied our previously developed WCST fMRI protocol (Monchi et al., 2001, 2004) in PD-MCI and PD non-MCI patients at two time points. We expected to find preserved activation of the cognitive cortico-striatal loop usually associated with planning a set-shift (Monchi et al., 2001, 2007; Nagano-Saito et al., 2014) in the PD non-MCI group over time. By contrast, we expected that PD-MCI patients would show a modification over time of activity beyond the cortico-striatal loops. Based on our previous studies showing that the hippocampus is one key region involved in cognitive function in PD patients (Nagano-Saito et al., 2004, 2005, 2014), we also predicted that recruitment of hippocampus activity at Time 1 would correlate with preserved cognition at Time 2.

## Materials and methods

### Subjects

Twenty-seven non-demented PD participants at stages I and II of Hoehn and Yahr were recruited for the study. All participants were assessed by the movement disorders neurologists (A-LL., SC., VS.) and diagnosed as PD with the UK brain bank criteria for idiopathic PD (Hughes et al., 1992). All patients were responsive to dopamine medication. Patients with other concurrent major neurological or psychiatric conditions were excluded. The demographic information of the patients is given in Table 1. All patients provided written informed consent, which was approved by the Research Ethics Committee of the Regroupement Neuroimagerie Québec.

**Table 1:** **PD patient demographics according to cognitive impairment group (MCI)**.

**Demographic data**	**PD non-MCI**	**PD with MCI**	**Difference *p-value***	**PD all**
**TIME 1**				
N	12	12	N/A	27
Age	58.2±5.4	62.25±5.6	0.941	60.3±5.5
Male/Female	6/6	8/4	0.248	15/12
Years since diagnosis	3.8±3.4	5.0±3.1	0.343	4.4±3.3
Education	14.6±2.5	13.8±3.2	0.495	14.1±3.0
Hand R/L/A	7/4/1	10/0/2	0.063	19/4/4
MoCA (off medication)	28.4±1.6	27.3±1.0	0.02	27.9±1.8
UPDRS (off medication)	27.2±8.9	26.9±8.3	0.935	26.6±8.1
BDI	6.0±4.2	11.3±6.1	0.046	9.0±5.7
**TIME 2**				
Age	59.9±5.5	64.0±5.5	0.083	61.9±5.5
Years since diagnosis	5.3±3.6	6.4±3	0.417	6.1±3.4
MoCA (off medication)	29.1±1.2	27±1.3	0.001	28±1.6
UPDRS (off medication)	27.3±6	26.9±8.3	0.918	26.6±7.5
BDI	7.8±3.9	11.3±6.1	0.119	9.3±5.4

The p-values indicate the group difference based on chi-squared test (sex and handedness) or student t-test (others). Legend: MoCA, Montreal cognitive assessment; UPDRS, Unified Parkinson's disease Rating Scale; BDI, Beck Depressive Inventory II. Individual MoCA scores are presented in Supplementary Table 3.

Participants were studied twice at 19.8 ± 2.7 months apart. In each session (at baseline Time 1 and follow-up at Time 2) they underwent a comprehensive neuropsychological assessment and fMRI during which they performed a computerized version of WCST (Monchi et al., 2001, 2004). Participants were asked not to take any dopaminergic medication at least 12 h prior to the sessions. Based on the neuropsychological assessment, participants were divided into two groups: those with MCI and those cognitively intact (non-MCI) at Time 1. Three patients with non-MCI at Time 1 turned into MCI at Time 2. Therefore, for the analyses to investigate the group difference, 24 subjects (mean age, 60.33 ± 5.8 years; 11 males and 13 females; 12 MCI, and 12 non-MCI) were included. For group-combined analyses, all 27 subjects (mean age 60.3 ± 5.5 years; 12 males and 15 females) were included.

Inclusion criteria for MCI were based on the Movement Disorder Society Task Force guidelines for Parkinson's disease (Level I and II) (Litvan et al., 2012), based on five cognitive domains (Supplementary Table 1) and were the same as our previous study (Nagano-Saito et al., 2014). Objective evidence of cognitive decline was set with performance >1.5 standard deviations below standardized mean of the same age-group on two or more subtests within a cognitive domain.

Demographically, no significant differences were observed between the groups with respect to age and the motor section of the Unified Parkinson's Disease Rating Scale at Time 1 and 2. Group demographic characteristics are listed in detail in Table 1.

### Neuropsychological assessment

A screening test, the Montreal Cognitive Assessment (MoCA) (Nasreddine et al., 2005), was administered at the beginning of each scanning session. The same comprehensive neuropsychological battery previously used in Jubault et al. (2009), Hanganu et al. (2013), Nagano-Saito et al. (2014), was performed by a licensed neuropsychologist (Dr. BMC) to assess five domains of cognition, attention and working memory, executive functions, language, memory and visuospatial functions. Details of the tasks used are given in Supplementary Table 1.

### Cognitive task during fMRI

A computerized version of the WCST (Monchi et al., 2001, 2004) was administered using stimulus presentation software. Participants were fully trained on the task prior to the scanning sessions. On each trial of the task, participants were asked to match a new test card to one of the four fixed reference cards based either on the color, shape, or the number of the stimuli in each reference card.

In WCST the classification rule was not given to the participant. Instead, s/he had to find it using the feedback (positive or negative) that followed each trial. On each experimental trial, participants had to find the proper classification rule, and apply it as long as a positive feedback preceded their response. A bright screen indicated a correct classification and a dark screen indicated an incorrect classification. On each control trial, the test card was identical to one of the four reference cards, therefore participants only had to select the twin reference card. On the control trials, the screen maintain its original brightness throughout the feedback period.

The first period of each trial started with the presentation of a new test card, at which point the participant choose one of the four reference cards. Response time was measured for each selection. The second period of each trial started as soon as the subject made a selection and consisted of feedback conveyed through a change in screen brightness lasting 2.3 s.

Each functional MRI run contained blocks of each of the four trial classifications (color, shape, number, and control) presented in random order. In WCST trial blocks, six consecutive correct matching responses were required before a change in classification rule could occur. Control blocks contained eight trials.

To evaluate the pattern of activation during the different stages of the WCST, four experimental and two control time periods were defined as follows: (1) Receiving negative feedback (RNF): the screen darkens indicating an incorrect response: a set-shift is therefore required and must be planned; (2) Matching after negative feedback (MNF): execution of the set-shift; (3) Receiving positive feedback (RPF): the screen brightens indicating a correct response: the current matching criterion must continue; (4) Matching after positive feedback (MPF): selection using the same classification rule as the previous trial; (5) Receiving control feedback (RCF): original screen brightness is maintained; (6) Matching with control feedback (MCF): select reference card identical to test card.

### fMRI scanning

Participants were scanned using the Siemens Tim Trio 3.0 T scanner at the Unité de Neuroimagerie Fonctionnelle of the Centre de Recherche de l'Institut Universitaire de Gériatrie de Montréal. Sessions began with high-resolution, T1-weighted, 3D volume acquisition for anatomical localization with resolution of 1 × 1 × 1 mm, followed by echoplanar T2^*^-weighted image acquisitions with blood oxygenation level-dependent (BOLD) contrast (echo time 30 ms; flip angle 90°; matrix size, 64 × 64 pixels; voxel size, 3.7 × 3.7 × 3.7 mm^3^). Functional images were acquired over five runs in a single session. Volumes were acquired continuously every 2.5 s, for a total 155 volumes within runs, and contained 36 slices.

### MRI data analysis

#### Contrast analyses

As was previously done with the same fMRI task, a General Linear Model data analysis was performed using fmristat (Monchi et al., 2001, 2004; Worsley et al., 2002; Jubault et al., 2009; Nagano-Saito et al., 2014). Briefly, the following contrasts were computed for each subject: 1. RNF vs. RPF: reflecting the planning of the set-shift, 2. MNF vs. MPF: reflecting the execution of the set-shift, 3. RPF vs. RCF: reflecting the maintaining of the set-shift, and 4. MPF vs. MCF: reflecting the matching according to the same rule. Next, the results of each run for each subject, were non-linearly transformed into standard proportional stereotaxic space (ICBM152 template) using anatomical MRI to template transformation parameters (Collins et al., 1994; Zijdenbos et al., 2002). In the second step, runs and subjects were combined using a mixed-effects linear model (Worsley et al., 2002). Both t-maps of inter-group (MCI and non-MCI of each time point) and within-group comparison (Time 1 vs. Time 2 of each group and combined groups) were generated. Statistical maps threshold was set at *p* < 0.05 correcting for multiple comparisons, yielding a threshold of *t* > 4.82 for a single voxel. Predicted peaks reaching *p* < 0.0001 (*t* > 3.87) uncorrected with a cluster size >40 mm^3^ assessed on the spatial extent of contiguous voxels are also reported and identified with an asterisk (*) in the tables. A region was predicted if it had been identified in our previous work using this task (Monchi et al., 2001, 2004; Jubault et al., 2009; Nagano-Saito et al., 2014) For within-group comparisons (Time 1 vs. Time 2), statistical maps threshold was set at *p* < 0.0001 (*t* > 3.87) uncorrected with a cluster size >40 mm^3^. Predicted peaks reaching *p* < 0.001 (*t* > 3.18) uncorrected with a cluster size >40 mm^3^ are reported.

Additionally, in order to evaluate the common neural regions solicited by both groups (PD-MCI and PD non-MCI), we performed a conjunction analysis using a conjunction null hypothesis with a threshold of *p* < 0.0001 for each group.

#### Correlation analysis

We wanted to separately address how individual cognitive performance ability affects the patterns of brain activity during the various stages of the WCST. To do this, we performed correlation analyses on the BOLD data while performing the WCST using cognitive scores of MoCA (Nasreddine et al., 2005) at the subject level. Secondly, in order to find which patterns of activity would be predictive of cognitive decline over time we correlated the BOLD data while performing the WCST at Time 1 with the difference of the MoCA scores between Time 1 and Time 2 at the subject level (Nasreddine et al., 2005). These analyses were performed across all participants combined (i.e., both MCI and non-MCI PD patients grouped). Finally, in order to look whether patterns of activity are associated with significant changes in performance of the WCST task, we correlated the BOLD data with the WCST accuracy and the WCST accuracy over time. These analyses were performed across all participants combined (i.e., both MCI and non-MCI PD patients grouped), at Time 1 and Time 2, separately.

Statistical maps threshold was set at *p* < 0.0001 (*t* > 3.87) uncorrected with a cluster size >40 mm^3^. Predicted peaks reaching *p* < 0.001 (*t* > 3.18) uncorrected are reported. A region was predicted if it had been identified in our previous work using the WCST. We especially expected involvement of the striatum, the thalamus, the hippocampus, and/or the PFC, because the activation of those regions was correlated with cognitive scores (Nagano-Saito et al., 2014).

## Results

### General cognitive scale measured by MoCA

The mean of MoCA is shown in the Table 1. A mixed-design repeated measures ANOVA (time × group) indicated main effect of group (*F* = 9.1, *p* = 0.006) but no effect of time and time × group interaction (*p* > 0.1).

### Performance of WCST

The error rates and reaction times for the task are shown in the Table 2. A mixed-design repeated measures ANOVA for the error rate and reaction time (task × time × group) indicated a main effect of task (*F* = 98.8, *p* < 0.001). No other main effects (including the group effect) or interaction was observed (*p* > 0.1). The mean reaction times are shown in Table 2. A mixed-design repeated measures ANOVA for the reaction time in control, after positive and after negative feedbacks (task × time × group) indicated a main effect of task (*F* = 29.9, *p* < 0.001), and time (*F* = 4.70, *p* = 0.041). A trend of interaction was observed for the time × task × group (*F* = 3.0, *p* = 0.059). No other significant effect was observed (*p* > 0.1).

**Table 2:** **Performance level of WCST**.

		**PD non-MCI**		**PD-MCI**	
		**Time 1**	**Time 2**	**Time 1**	**Time 2**
**Error rate (%)**	Control	2.7±3.0	3.2±2.7	2.8±4.3	4.5±4.2
	WCST	13.3±6.5	13.8±8.9	19.2±6.9	15.9±7.1
**Reaction time (sec)**	Control	2.45±0.76	2.58±0.73	2.50±0.56	3.17±1.25
	After positive feedback	2.64±0.64	2.89±0.74	2.88±0.55	3.16±1.15
	After negative feedback	3.05±0.53	3.39±0.81	3.41±0.86	3.85±1.80

#### Imaging analysis

Our previous study at Time 1 indicated no significant difference of other contrasts in the intergroup comparison. Thus, we selectively reported the comparison RNF vs. RPF (planning the set shift) and MNF vs. MPF (executing the set-shift), because our previous study at Time 1 indicated no significant difference of other contrasts in the intergroup comparison (Nagano-Saito et al., 2014).

#### Planning the set-shift

Similar to our previous study (Nagano-Saito et al., 2014), when planning the set shift at Time 1, the PD non-MCI group demonstrated significant activation in the PFC, precuneus, parietal cortex, occipital cortex and cerebellum (Table 3). Activation was also observed in the caudate when a lower threshold was used (*p* < 0.001 uncorrected). At Time 2, this group demonstrated significant activations in the posterior PFC, ventrolateral PFC, medial PFC, occipital, and parietal cortices. Activation was again observed in the caudate when a lower threshold was used (*p* < 0.001 uncorrected). Longitudinal comparison (Time 1 vs. Time 2) indicated decreased activity at Time 2 in the occipital area and cerebellum hemisphere, and increased activity at Time 2 in the right parietal lobe and the posterior prefrontal frontal cortex (Supplementary Table 2).

**Table 3:** **Significant activations in the contrasts for RNF minus RPF, and MNF minus MPF**.

**Anatomical Area**		**Non-MCI**				**MCI**				**Non-MCI and MCI (conjunction)**			
		**Time 1**		**Time 2**		**Time 1**		**Time 2**		**Time 1**		**Time 2**	
		**XYZ**	***t***	**XYZ**	***t***	**XYZ**	***t***	**XYZ**	***t***	**XYZ**	***t***	**XYZ**	***t***
**RNF–RPF**													
DLPFC	L	−40, 38, 8	3.95^*^										
	R					40, 28, 18	4.49^*^	42, 24, 18	4.46^*^				
								44, 34, 14	4.34^*^				
VLPFC	L	−32, 26, 0	5.01										
	R	32, 24, 2	4.82	30, 26, −6	4.71^*^			32, 28, −2	4.36^*^			20, 28, −4	7.92
mPFC	L			−6, 28, 40	4.19^*^			−8, 26, 40	4.29^*^			−16, 26, 42	8.38
	R			6, 24, 36	4.12^*^								
pPFC	L	−42, 6, 32	4.69^*^	−40, 12, 24	4.64^*^			−42, 10, 28	4.52^*^	−38, 10, 26	4.98	−40, 10, 30	7.35
	R	44, 8, 20	4,37^*^	38, 6, 24	5.11	44, 8, 24	4.28^*^	44, 14, 30	4.8^*^	40, 14, 24	6.07	44, 10, 28	8.34
		40, 14, 24	4.26^*^			38, 18, 22	4.45^*^						
Parietal	L	−30, −70, 28	5.77	−32, −60, 38	4.67^*^								
	R	32, −74, 20	5.38	32, −60, 50	4.17			30, −70, 32	5.34				
								26, −64, 56	3.99^*^				
Precuneus	L	−10, −74, 48	4.29^*^										
Occipital	L	−22, −82, −14	6.98	−24, −78, −8	4.34^*^								
				−40, −84, 14	4.5^*^								
	R	40, −82, 6	4.11^*^	36, −84, 12	5.35	42, −80, 12	4.01^*^	40, −84, 12	5.28	40, −82, 12	5.33	38, −84, 12	9.71
		30, −60, −12	6.73					20, −66, 26	4.58^*^			26, −60, −12	7.39
Cerebellum	L	−10, −80, −28	4.99										
		−32, −66, −32	4.62^*^										
	R	8, −80, −26	4.42^*^										
		30, −70, −30	4.31^*^										
Caudate	L	−14, 20, −4	3.77^**^										
	R			16, 16, −6	3.48^**^								
**MNF**−**MPF**													
DLPFC	L	−46, 40, 28	5.23	−46, 30, 34	5.01								
	R	42, 40, 32	4.74^*^	42, 30, 36	4.54^*^								
				46, 40, 22	5.2								
PMC	L	−26, 30, 58	4.08^*^										
	R	24, 24, 58	4.78^*^										
VLPFC	L	−32, 26, −8	4.64^*^										
	R	32, 26, −8	4.04^*^										
mPFC	L			−2, 16, 50	3.95^*^								
	R	0, 28, 44	5.63	6, 24, 44	4.62^*^								
aPFC	L	−36, 54, 12	5.02			−32, 52, 8	4.4^*^			−32, 52, 8	6.33		
	R	38, 56, 18	4.5^*^	32, 60, 6	4.15^*^								
		38, 50, 6	4.21^*^										
		42, 52, 14	4.03^*^										
Parietal	L	−34, −54, 40	4.84^*^	−34, −62, 38	5.44			−30, −54, 44	4.33^*^			−32, −56, 40	6.01
								−62, −66, 30	4.24^*^				
	R	36, −62, 60	4.69^*^	22, −70, 60	4.23^*^								
		50, −44, 54	4.35^*^	46, −62, 52	4.1^*^								
Precuneus	L			−4, −76, 48	4.33^*^			−12, −74, 56	4.82^*^			−4, −78, 50	5.98
	R	2, −68, 44	4.48^*^	14, −78, 56	4.31^*^								
Occipital	L	−26, −96, 0	5.5	−30, −84, −12	4.05^*^								
	R	34, −92, 0	5.75			−28, −84, 34	5.06	28, −80, −18	4.13^*^				
								32, −74, 24	4.13^*^				
Cerebellum	L	−40, −76, −46	4.37^*^					−32, −76, −16	4.46^*^				
								−6, −78, −26	4.29^*^				
								−18, −84, −22	4.1^*^				
	R	24, −86, −20	4.74^*^			30, −64, −32	4.76^*^						

*Legend. Results presented at t > 4.82; p < 0.05 corrected for multiple comparisons*.

* *predicted regions (t > 3.87; p < 0.0001)*.

** *trends (t > 3.18; p < 0.001). t, t-value; L, left; R, right; DLPFC, dorsolateral prefrontal cortex (BA 46, 9/46); pPFC, posterior PFC (BA 6, 8, 44); VLPFC, ventrolateral prefrontal cortex and insula (BA 47/12/13); aPFC, anterior prefrontal cortex (BA 10); mPFC, medial prefrontal cortex (BA 6, 8, 32); Parietal, parietal cortex (BA 40, 7); Precuneus, precuneus cortex (BA 40, 7); Occipital, occipital or striate/ extrastriate cortices (BA 17, 18, 19); PMC, premotor area (BA 6)*.

By contrast, the PD-MCI group demonstrated significant activation peaks in the dorsolateral PFC, posterior PFC, and the occipital cortex. At Time 2, PD-MCI revealed significant activation peaks in the dorsolateral PFC, ventrolateral PFC, medial PFC, posterior PFC, posterior parietal cortex, and visual occipital area (Table 3). Longitudinal analyses showed decreased activity at Time 2 in the cerebellum and increased activity at Time 2 in the visual occipital cortex (Supplementary Table 2).

Conjunction analysis over both groups revealed significant activations in the posterior PFC and occipital cortex at Time 1, as well as significant activation in the ventrolateral PFC, posterior PFC and occipital cortex at Time 2 (Table 2, RNF-RPF).

**Figure 1:**
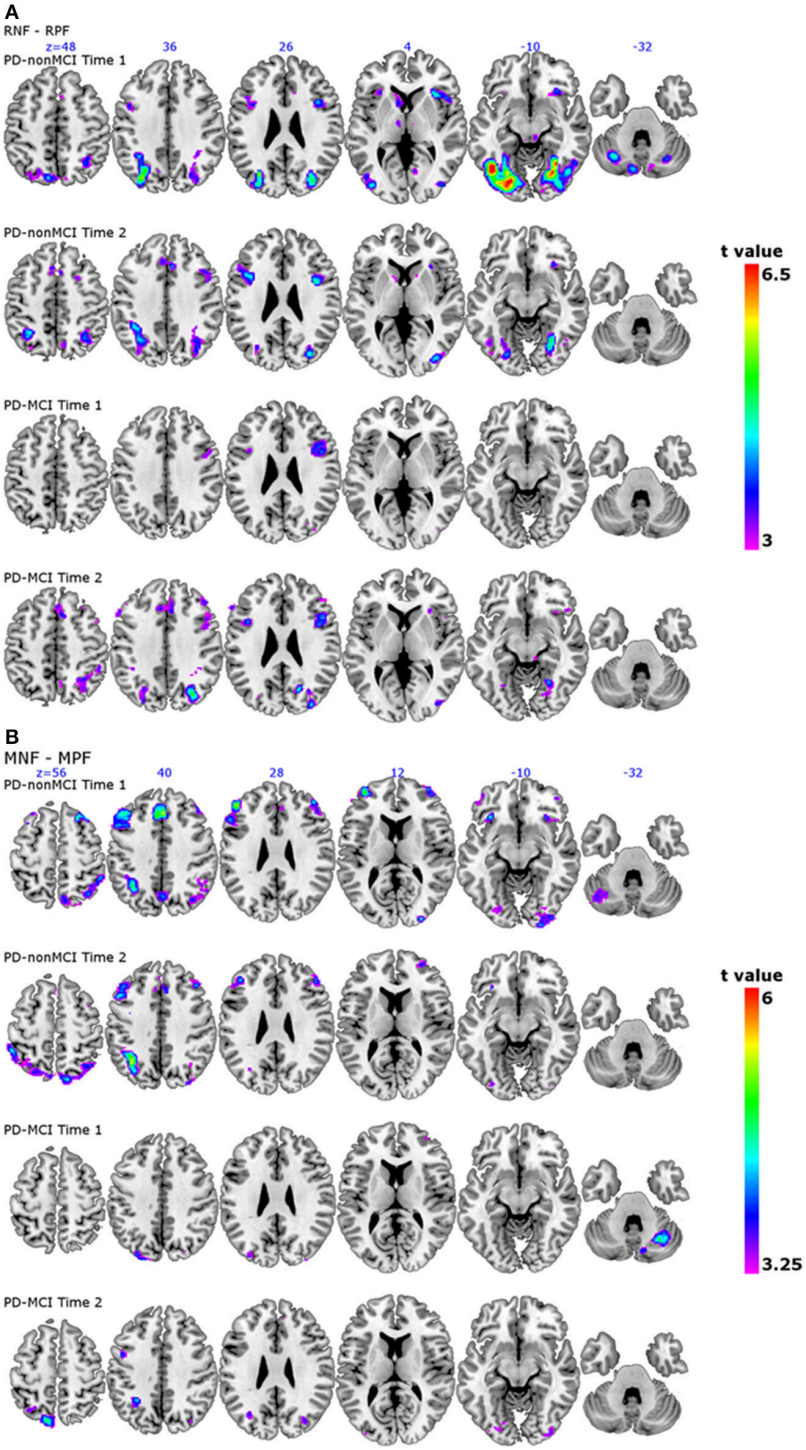
**Patterns of activation in PD-nonMCI and PD-MCI at time 1 and time 2 separately. (A)** The contrast receiving negative feedback vs. receiving positive feedback, corresponding to planning the set-shift, is shown. For each group at each time point, slices are shown at ICBM co-ordinates *Z* = 48, *Z* = 36, *Z* = 26, *Z* = 4, *Z* = −10, and *Z* = −32. **(B)** The contrast matching following negative feedback vs. matching following positive feedback, corresponding to executing the set-shift is shown. For each group at each time point, slices are shown at ICBM co-ordinates *Z* = 56, *Z* = 40, *Z* = 28, *Z* = 12, *Z* = −10, and *Z* = −32.

The localisation of the observed peaks are shown in Figure 1A. The results of the longitudinal comparison (Time 1 vs. Time 2) with all the subjects (*n* = 27) is also shown in the Supplementary Table 2.

#### Executing the set-shift

When executing the set-shift, the non-MCI group at Time 1 revealed significant activations in the dorsolateral PFC, premotor cortex, ventrolateral PFC, medial PFC, anterior PFC, parietal cortex, precuneus, the visual occipital areas, and cerebellum (Table 3). The pattern was similar to our previous study (Nagano-Saito et al., 2014). At Time 2, the PD non-MCI group revealed significant activation in the dorsolateral PFC, medial PFC, anterior PFC, parietal cortex bilaterally, precuneus and occipital visual area (Table 3). Longitudinally, there was reduced activation in the cerebellum and increased activity in the precuneus at Time 2 compared to Time 1 (Supplementary Table 2).

By contrast, the PD-MCI group demonstrated activation peaks in the middle occipital cortex, occipital cortex and cerebellum at Time 1, while at Time 2, positive significant activations were revealed in the parietal lobe, precuneus, visual occipital areas and cerebellum (Table 3). Longitudinally there were no significant differences between Time 1 and Time 2 in the PD-MCI group (Supplementary Table 2).

Conjunction analysis over both groups revealed significant activations in the anterior PFC at Time 1 and activations in the parietal cortex and precuneus at Time 2 (Table 2, MNF-MPF).

The localisation of the observed peaks are shown in Figure 1B. The results of the longitudinal comparison (Time 1 vs. Time 2) with all the subjects (*n* = 27) is also shown in the Supplementary Table 2.

#### Correlation analysis

When planning the set-shift, no significant correlation was observed with the MoCA scores at Time 1. A significantly positive correlation was observed in the medial PFC at Time 2, overlapping with the more activated area at Time 2 (Table 4). When executing the set-shift, significant correlation was observed in the striatum and the left thalamus at Time 1 as well as with the precuneus at Time 2.

**Table 4:** **Correlation between regions in all PD patients with MoCA score and WCST accuracy (Time 1 vs. Time 2)**.

**Anatomical Area**		**XYZ**	***t***
**CORRELATION BETWEEN REGIONS AND THE MOCA SCORE**			
**MoCA score and fMRI Time 1**			
**MNF–MPF**			
Thalamus	L	−18, −20, 10	3.61^*^
Striatal	L	−8, −54, 0	5.16
**MoCA score and fMRI Time 2**			
**RNF–RPF**			
mPFC	L	−6, 38, 34	3.99
**MNF–MPF**			
Precuneus	L	−6, −82, 46	4.63
**MoCA score over time, fMRI Time 1**			
**RNF-RPF**			
Internal Capsule	R	28, −22, 6	4.56
Thalamus	L	−12, −24, 8	4.57
	R	16, −26, 8	4.31
Hippocampus	L	−34, −32, −10	3.48^*^
	R	36, −30, −12	3.54^*^
**MNF-MPF**			
mPFC	R	4, 34, 48	3.25^*^
**CORRELATION BETWEEN REGIONS AND THE WCST ACCURACY**			
**Accuracy and fMRI Time 1**			
**RNF–RPF**			
Ventral striatum	L	−20, 14, −8	5.49
	R	20, 14, −6	4.35
VLPFC	L	−30, 26, 0	3.7^*^
Occipital	L	−46, −62, −10	4.5
**MNF–MPF**			
mPFC	L	−8, 24, 38	4.41
	R	8, 36, 34	5.14
DLPFC	L	42, 18, 26	3.57^*^
	R	40, 22, 40	3.9
Caudate	R	12, 2, 16	3.49^*^
Hippocampus	R	24, −22, −16	3.31^*^
**Accuracy and fMRI Time 2**			
**RNF–RPF**			
VLPFC	R	32, 22, 8	3.66^*^
PMC	L	−46, 6, 22	3.25^*^
Parietal	R	36, −48, 36	4.08
Caudate	L	−16, 16, 8	3.44^*^
**Accuracy over Time, fMRI Time 1**			
**RNF-RPF**			
aPFC	L	−28, 52, −4	4.43
**MNF-MPF**			
DLPFC	R	38, 18, 32	3.54^*^
mPFC	L	−10, 20, 32	4.11
Occipital	L	−58, −58, 12	5.84
Parietal	L	−46, −64, 35	4.19
Precuneus	L	−6, −42, 36	5.29
Hippocampus	R	22, −20, −14	3.91

*Legend. Results presented at t > 3.87; p < 0.0001, uncorrected*;

* *indicates predicted regions (t > 3.18; p < 0.001 uncorrected). t, t-value; L, left; R, right; DLPFC, dorsolateral prefrontal cortex (BA 46, 9/46); VLPFC, ventrolateral prefrontal cortex and insula (BA 47/12/13); aPFC, anterior prefrontal cortex (BA 10); mPFC, medial prefrontal cortex (BA 6, 8, 32); Parietal, parietal cortex (BA 40, 7); Precuneus, precuneus cortex (BA 40, 7); Occipital, occipital or striate/ extrastriate cortices (BA 17, 18, 19); PMC, premotor area (BA 6)*.

**Figure 2:**
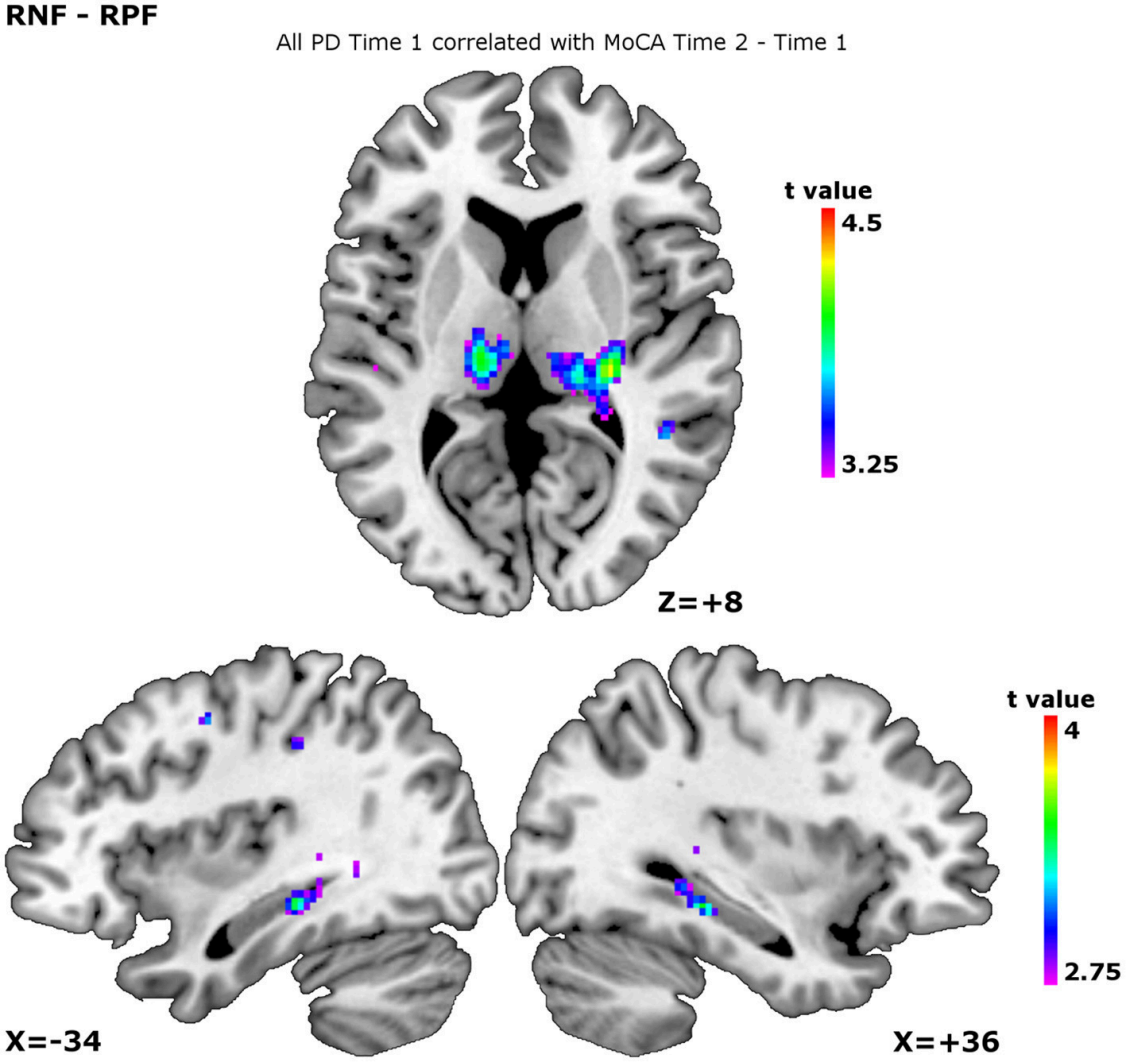
**Patterns of activity over all participants at time 1 correlating with MoCA evolution Time 2 vs. Time 1**. The correlation patterns are shown for the contrast receiving negative feedback vs. receiving positive feedback, corresponding to planning the set-shift. The higher the peaks, the less MoCA decline. This was observed in the thalamus, show on the slice at ICBM co-ordinate *Z* = +8, the left hippocampus, shown on the slice at ICBM co-ordinate *X* = −34, and the right hippocampus, shown on the slice at ICBM co-ordinate *X* = +36.

When we added the MoCA decline across the time points as a confounder, significant negative correlations were observed in the internal capsule, thalamus, and hippocampus during planning the set-shift (Table 4, Figure 2). During executing the set-shift, significant negative correlations were observed in the medial PFC (Table 4). They indicate that less cognitive decline occurs as more of these regions are solicited.

Correlations with WCST accuracy were also depicted. Planning the set-shift showed significant correlations with the ventral striatum, ventrolateral PFC and occipital area at Time 1, as well as with ventrolateral PFC, premotor cortex, parietal, and caudate at Time 2. Executing the set-shift showed significant correlations between the WCST accuracy and activations in the medial PFC, dorsolateral PFC, caudate, hippocampus at Time 1, but no correlation were shown at Time 2.

When WCST accuracy over time was correlated with BOLD at Time 1, planning the set-shift showed significant correlations with the anterior PFC, while executing the set-shift showed correlations with dorsolateral PFC, medial PFC, occipital cortex, parietal, precuneus, and hippocampus, indicating that greater activity in these regions at Time 1 is indicative of preserved performance on the WCST over time.

## Discussion

We longitudinally followed up the brain neural activation of non-demented PD patients undergoing an fMRI session while performing the WCST with different levels of cognitive impairment. As predicted, the PD non-MCI and PD-MCI groups were different in their patterns of activity-change over time when performing the WCST.

### PD non-MCI patterns over time

#### Patterns of preserved activity

At both time points, significant activation was observed in the frontal and parietal cortex, with a trend in the caudate, during planning the set-shift. Fronto-striatal activation in the cognitive loop has been reported to be preserved in non-MCI PD patients, but affected in MCI patients (Lewis et al., 2003; Ekman et al., 2012; Nagano-Saito et al., 2014: Table 3). Thus, our observation may indicate relatively preserved function in the cognitive loop at Time 2 in the non-MCI PD patients. This is in agreement with another longitudinal study showing stable activity across time-points in non-MCI patients with the n-back working memory task (Ekman et al., 2014). During the execution of the set-shift, significant activation was also preserved in the frontal and parietal cortex (Table 3). However, overall, the activation was relatively weak at Time 2, and intra-group comparison did not reach significance. Considering the fact that the activated regions during this period could correspond to the motor-loop (Monchi et al., 2004, 2007) this relatively weak activation may reflect the disease progress affecting more motor-related function in non-MCI PD patients. Actually, slower RT was observed at Time 2 in non-MCI PD patients (Table 2), without a change in accuracy in the performance of the WCST.

#### Regions with decreased patterns of activity over time

Activation in the occipital visual cortex during planning the set-shift and the cerebellum during planning and executing the set-shift, was reduced during the time course, between Time 1 and Time 2 (Supplementary Table 2). It has been reported in non-demented PD patients that lowered regional cerebral metabolic rates in the occipital area correlate with the motor dysfunction (Bohnen et al., 1999) We also previously reported lower regional cerebral metabolic rates for glucose in the occipital area accompanied by dopaminergic availability in the striatum, along with motor dysfunction in non-demented PD patients (Nagano-Saito et al., 2004) Simultaneously, cerebellar activity is likely to contribute to pathophysiological change underlying PD (Martinu and Monchi, 2013) Thus, the reduced activation in the occipital area and cerebellum may reflect remote effects of the progress of the disease on dopamine projections in the striatum. However, they might also reflect a learning effect with reduction of visual attention and motor effort at Time 2. A longitudinal study with healthy volunteers would help to understand these observations.

### PD-MCI patterns over time

#### Patterns of increased activity

During planning the set-shift, PD-MCI revealed significantly increased activation in the frontal, parietal and occipital areas at Time 2 (Table 3). These regions were overlapped with the activation pattern of PD non-MCI. PD patients have been reported to often have fluctuations in non-motor functions (Witjas et al., 2002) This might explain the relatively higher performance at Time 2 observed in this group of patients. Actually, the error rate was smaller with MCI patients across time, although it did not reach significance. Nevertheless, RT became slower at Time 2. This may indicate that the relay of information from the cognitive loop to motor loop could not be recovered.

During executing the set-shift, more activation was observed in the parietal areas, precuneus and cerebellum, but not in the frontal regions at Time 2 (Table 4). Thus, the recovered activation is not likely to be supported by the cortico-striatal loops, but by alternative brain circuit.

### Medial PFC and precuneus patterns of activation in both groups

We observed significant activation in the medial PFC during planning the set-shift in both groups and in the conjunction analyses only at Time 2 (Table 3). Moreover, in the group of all patients during planning a set-shift, the activity in the medial PFC showed a positive correlation with the MoCA scores at Time 2 and preserved WCST accuracy score over time (Table 4). In addition, when executing the set-shift, increased activity in the medial PFC correlated with preserved cognition over time (as measured by the MoCA), with WCST accuracy at Time 1 and with preserved WCST accuracy score over time (Table 4). The mPFC is considered to play an important role in learning association between events and in linking adaptive responses (Euston et al., 2012). The medial PFC activates both during externally guided and internally guided decision making tasks (Nakao et al., 2012). The recovery of the mPFC activity at Time 2 could therefore help in relating the cognitive processes required to select a response to the actual motor selection.

Another region that was recruited during executing the set-shift at Time 1 in PD non-MCI only and at Time 2 in both groups which was confirmed by the conjunction analyses at Time 2 (Table 3) was the precuneus. In the all patients group the strength of the activation in the precuneus during executing the set-shift showed a significantly positive correlation with the MoCA scores at Time 2 and the preserved WCST accuracy over time (Table 4). This shows the importance of this region's function for cognition. The precuneus, as a part of the parietal cortex (Vogt et al., 2001), functionally connects to the medial PFC and superior frontal cortex (Laird et al., 2009; Margulies et al., 2009), and is considered to be involved in reaching to visual targets (Bernier and Grafton, 2010). More generally, precuneus and medial PFC are components of the dorsomedial motor stream. (Rizzolatti and Matelli, 2003; Binkofski and Buxbaum, 2013) Interestingly, in PD non-MCI the posterior PFC, which is considered as a part of the dorsomedial motor stream, was higher at Time 2, compared to Time 1, although the more anterior PFC showed tendency of decreasing of activity. Previous studies reported decreased resting state connectivity in bilateral PFC (as part of the dorsomedial motor network) and fronto-parietal areas in PD-MCI patients (Amboni et al., 2015; Baggio et al., 2015), along with a ordered connectivity reduction (HC > PD, non-MCI > PD-MCI). Thus, our results for the precunes and the medial PFC activation may reflect increased recruitment of the dorsomedial motor stream, compensating the cortico-striatal loop, and allowing a better performance as shown by the correlations with WCST accuracy.

### Patterns of activity predictive of cognitive evolution

When planning the set-shift, increased activity in the thalamus and the hippocampus correlated with preserved cognition over time as measured by the MoCA (Table 4, Figure 2). Furthermore, when executing the set-shift the increased activity in the hippocampus correlated with preserved WCST performance over time (Table 4).

We recently reported that cortical thinning occurs significantly faster overtime in the medial temporal lobe in PD-MCI vs. PD non-MCI, suggesting that temporal lobe atrophy could be used as a predictor of dementia in PD (Hanganu et al., 2014). Carbon et al. (2010), performed a longitudinal PET study in PD patients and controls, where participants performed a visual sequence-learning task (Carbon et al., 2010). The researchers observed that while the hippocampus was not significantly solicited in controls during the task, the PD patients whose performance did not decline between the two time points (i.e., the best performers) showed significant increase in rCBF in the hippocampus over time, as compared to the lower performers. They suggested that a compensatory hippocampal activation response may be a specific functional indicator of incipient cognitive decline in non-demented PD patients at early disease stages. Our present results are in agreement with this notion.

We have observed decreased thalamus activity in PD vs. controls in the context of two different set-shifting tasks (Monchi et al., 2001, 2007) hence, the thalamus activity level correlating with preserved cognition may reflect the importance of the preserved function of cognitive cortico-striatal loop. Furthermore, connectivity between the hippocampus and the posterior parts of the thalamus has been observed in animals (Herkenham, 1978; Wouterlood et al., 1990; Vertes et al., 2006) and in humans (Behrens et al., 2003). Our previous fMRI study, which examined the same task in healthy volunteers, showed that hippocampal deactivation was less accompanied by extensive activation in the thalamus during a set-shift, when dopamine was lowered (Nagano-Saito et al., 2008). A recent voxel based morphometry study reported reduced thalamic and hippocampal gray matter intensity in PD-MCI vs. PD non-MCI (Chen et al., 2016). Thus, the functional integrity of the thalamus, possibly connecting to the hippocampus, might be an important marker of cognitive preservation in PD.

## Conclusion

In conclusion, our results show differential neural changes in PD-MCI patients as compared with the PD non-MCI group over time. They indicate that, as long as the cognitive cortico-striatal loops are preserved, the circuit seems to be recruited. However, when the cortico-striatal loops cannot perform the demanded functional task, the extra circuits, including the mPFC, and the precuneus, could be recruited. Moreover, hippocampal compensation is used to maintain cognitive abilities over time. More work in this area is warranted to determine the precise phenotypes of such sub-groups of patients.

## Author contributions

Research project: Conception: OM. Organization: BM, CB, AL, VS, and SC. Execution: CD, BM, AH, CB, AL, VS, and SC. Statistical Analysis: Design: OM and AN. Execution: MA and AN. Review and Critique: OM and AN. Manuscript: Writing of the first draft: MA and AN. Review and Critique: OM, AN, and AH.

## Funding

This work was supported by a Canadian Institutes of Health Research grant (MOP-81114), a psychosocial grant from the Parkinson Society Canada, the Canada Research Chair in non-motor symptoms of Parkinson's disease, and the Tourmaline Oil Chair in Parkinson's disease to OM.

### Conflict of interest statement

The authors declare that the research was conducted in the absence of any commercial or financial relationships that could be construed as a potential conflict of interest.
