# Supplementary material for: Patterns of Longitudinal Neural Activity Linked to Different Cognitive Profiles in Parkinson's Disease
Source: Front Aging Neurosci. 2016 Nov 23;8:275. doi: 10.3389/fnagi.2016.00275 (PMC5120116; doi:10.3389/fnagi.2016.00275)
Supplement: Supplementary file 1 [file Table1.DOCX]

**Supplementary Table 1.**

Neuropsychological test battery according to cognitive domain

| **Cognitive domain** | **Test** |
| --- | --- |
| **Attention and working memory** | Trail Making Test Part A (Reitan and Wolfson, 1985)  Digit span test (Wechsler, 1997)  Stroop color-word test, reading and color naming parts (Golden and Freshwater, 1998) |
| **Executive function** | Tower of London (Culbertson and Zillmer, 2005)  Brixton (Burgess and Shallice, 1997)  MEC, orthographic verbal fluency subtest (Joanette et al., 2004)  Trail Making Test Part B (Reitan and Wolfson, 1985)  Stroop color-word test, interference part (Golden and Freshwater, 1998) |
| **Language** | Wechsler Abbreviated Scale of Intelligence, vocabulary subtest (Wechsler, 1999)  Boston Naming (Kaplan et al., 1983)  MEC, semantic verbal fluency subtest (Joanette et al., 2004) |
| **Memory** | Rey Auditory Verbal Learning Test (Schmidt, 1996)  Wechsler Memory Scale 3^rd^ ed., logical memory subtest (immediate and delayed recalls) (Wechsler, 1997) |
| **Visuo-spatial function** | Hooper Visual Organization Test (Hooper, 1958)  Clock-drawing subtest of the MoCA, evaluated by scores of Schulman et al. (Shulman et al., 1993; Shulman, 2000; Nasreddine et al., 2005) |

Legend. MEC, Montreal Evaluation of Communication protocol; MoCA, Montreal Cognitive Assessment Scale.
