# Supplementary material for: Patterns of Longitudinal Neural Activity Linked to Different Cognitive Profiles in Parkinson's Disease
Source: Front Aging Neurosci. 2016 Nov 23;8:275. doi: 10.3389/fnagi.2016.00275 (PMC5120116; doi:10.3389/fnagi.2016.00275)
Supplement: Supplementary file 2 [file Table2.DOCX]

**Supplementary Table 2.** Activation change in the brain over time (Time 1 vs Time 2)

| **Anatomical Area** | | **Non-MCI** | | **MCI** | | **All** | |
| --- | --- | --- | --- | --- | --- | --- | --- |
|  |  | XYZ | t | XYZ | t | XYZ | t |
| ***RNF - RPF*** | | | | | | | |
| **Time 1 > Time 2** | | | | | | | |
| Occipital | L | -18, -96, -6 | 3.63* |  | | -30, -92, -8 | 4.21 |
|  | R | 2, -92, 22 | 3.72* |  | | | |
| Cerebellum | L | -12, -84, -22 | 3.60* | -34, -88, -24 | 3.64* | -38, -82, -20 | 3.99 |
|  |  |  |  |  |  | -44, -74, -42 | 3.89 |
|  | R |  | | | | 38, -78, -44 | 3.54 |
| M1 | L |  | | | | -52, -22, 58 | 3.82 |
| **Time 1 < Time 2** | | | | | | | |
| pPFC | R | 34, 18, 58 | 4.56 |  | | 32, 18, 54 | 4.67 |
| mPFC | R |  | | | | 2, 32, 36 | 3.51* |
| Parietal | R | 22, -68, 64 | 3.90 |  | | 34, -64, 58 | 4.61 |
| Occipital | R |  | | 12, -62, 18 | 4.19* |  | |
| putamen/ Insula | L |  | | | | -32, 0, -6 | 4.02 |
|  | R |  |  |  |  | 40, 10, -4 | 4.10 |
| Cerebellum (Vermis) | L |  | | | | -8, -50, -30 | 4.32 |
|  | R |  |  |  |  | 4, -48, -32 | 3.98 |
| ***MNF - MPF*** | | | | | | | |
| **Time 1 > Time 2** | | | | | | | |
| Cerebellum | L | -48, -76, -24 | 3.53* |  | | | |
|  | R | 28, -92, -26 | 3.62* |  |  |  |  |
| **Time 1 < Time 2** | | | | | | | |
| Precuneus | R | 0, -46, 70 | 3.77* |  | | | |
| Cingulate | L |  | | | | -10-18, 44 | 4.40 |

Legend. Results presented at t>3.87; p<0.0001, uncorrected; * indicates predicted regions (t>3.18; p<0.001 uncorrected). t= t-value; L= left; R= right; DLPFC= dorsolateral prefrontal cortex (BA 46, 9/46); pPFC= posterior PFC (BA 6, 8, 44); mPFC= medial prefrontal cortex (BA 6, 8, 32); Parietal= parietal cortex (BA 40, 7); Precuneus= precuneus cortex (BA 40, 7); Occipital= occipital or striate/ extrastriate cortices (BA 17, 18, 19); M1=primary motor cortex.
