# Supplementary material for: Patterns of Longitudinal Neural Activity Linked to Different Cognitive Profiles in Parkinson's Disease
Source: Front Aging Neurosci. 2016 Nov 23;8:275. doi: 10.3389/fnagi.2016.00275 (PMC5120116; doi:10.3389/fnagi.2016.00275)
Supplement: Supplementary file 3 [file Table3.DOCX]

**Supplementary Table 3.** Individual MoCA values for all participants.

| **PD non-MCI** | | **PD-MCI** | |
| --- | --- | --- | --- |
| **Time 1** | **Time 2** | **Time 1** | **Time 2** |
| 26 | 30 | 26 | 26 |
| 29 | 30 | 26 | 27 |
| 29 | 29 | 26 | 27 |
| 29 | 28 | 28 | 26 |
| 30 | 30 | 28 | 27 |
| 30 | 29 | 27 | 28 |
| 30 | 30 | 28 | 26 |
| 29 | 30 | 28 | 30 |
| 29 | 28 | 28 | 25 |
| 26 | 26 | 28 | 26 |
| 26 | 29 | 28 | 28 |
| 28 | 30 | 29 | 28 |
